# Validation of the fear of cancer recurrence inventory-short form in long-term colorectal cancer survivors

**DOI:** 10.1186/s41687-026-01051-y

**Published:** 2026-04-18

**Authors:** Katrine Ingeman, Tina Birgitte Wisbech Carstensen, Allan Ben Smith, Sébastien Simard, Lisbeth Frostholm, Eva Ørnbøl, Kaare Bro Wellnitz, Johanne Dam Lyhne

**Affiliations:** 1 Department of Functional Disorders, Aarhus University Hospital, Aarhus, Denmark; 2 Department of Clinical Medicine, Aarhus University, Aarhus, Denmark; 3 The Daffodil Centre, A Joint Venture with Cancer Council NSW, The University of Sydney, Sydney, Australia; 4 South West Sydney Clinical Campuses, Faculty of Medicine and Health, UNSW Sydney, Sydney, Australia; 5 Université du Québec à Chicoutimi (UQAC), Chicoutimi (Qc), Canada; 6 University Hospital of Southern Denmark, Vejle, Denmark

**Keywords:** Fear of cancer recurrence, Validation, Screening, Colorectal cancer, Survivorship care, Psycho-oncology

## Abstract

**Background:**

The Fear of Cancer Recurrence Inventory-Short Form (FCRI-SF) is commonly used to categorize the severity of fear of cancer recurrence (FCR) defined as fear, worry, or concern about cancer returning or progressing. Evaluating the FCRI-SF factor structure and the clinical cut-off score in specific cancer populations and stages of survivorship is essential to determine its generalizability as a screening tool for clinical FCR. This study aimed to (1) examine the factor structure of the FCRI-SF, and (2) evaluate the clinical cut-off score in long-term colorectal cancer survivors without recurrence.

**Methodology:**

Data were drawn from a national cohort study on late effects after colorectal cancer and a randomized trial evaluating an online FCR intervention. Exploratory and confirmatory factor analyses were conducted to examine the latent structure of the FCRI-SF. The optimal theoretical cut-off was assessed using weighted logistic regression to estimate sensitivity and specificity.

**Results:**

A total of 5,515 survivors completed the FCRI-SF, and 334 underwent clinical FCR assessment. Factor analyses indicated a one-factor solution, though model fit indices were suboptimal. Item 5 showed poor psychometric performance, and item 9 had a bimodal response pattern. The optimal theoretical cut-off score for identifying clinical FCR was 22.

**Conclusions:**

The FCRI-SF is a suitable tool for assessing FCR in long-term colorectal cancer survivors, but minor revisions to item 5 and item 9 may improve its psychometric properties.

**Supplementary Information:**

The online version contains supplementary material available at 10.1186/s41687-026-01051-y.

## Introduction

Fear of cancer recurrence (FCR) has been recognized as a pressing, unmet psychosocial need among cancer patients [1–3]. FCR is defined as fear, worry, or concern about cancer returning or progressing [4]. Experiencing some degree of FCR is normal in cancer survivors and can be adaptive when it promotes healthy lifestyle and adherence with doctor’s recommendations. However, high levels of FCR can severely impact daily functioning and quality of life, primarily due to persistent intrusive thoughts about recurrence. Moreover, without appropriate intervention, FCR may persist for many years after cancer treatment [1, 5–7]. Characteristics of clinical FCR are identified as high levels of preoccupation with symptoms, high levels of persistent worry about recurrence, and hypervigilance to bodily symptoms [8].

Different measures of FCR exist [9, 10], but one of the psychometrically strongest and most frequently used is the Fear of Cancer Recurrence Inventory (FCRI) which consists of 42 items divided into seven subscales (Triggers, Severity, Psychological Distress, Coping Strategies, Functioning Impairments, Insight, and Reassurance) [11]. The severity subscale is also recognized as the FCRI-Short Form (FCRI-SF) and is widely used as a screening tool for FCR in research contexts. Previously established cut-off scores on the FCRI-SF include a score of 13 for general screening purposes (i.e., to identify potential presence of FCR) [11] and a score of 22 for identifying clinical cases of FCR likely needing treatment [12].

Clinical or severe FCR is estimated to occur in approximately 19% of all cancer survivors across diagnosis [11–13]. FCR is generally more prevalent in younger age groups [1, 13, 14] but has also been recognized as a key concern for older survivors (> 64 years) [6]. In the current sample of long-term colorectal cancer survivors (defined as ≥ 5 years since cancer diagnosis with no recurrence and no residual disease), applying the cut-off score of 22 on the FCRI-SF, 5% reported clinical FCR [15].

When using the FCRI-SF for identifying FCR requiring treatment, some authors have questioned if the items in the FCRI-SF adequately reflects the consensus-based characteristics of clinical FCR [2, 16, 17]. During this discussion, the FCRI-SF has been criticized for including aspects that are not regarded as reflective of FCR severity [8, 17]. One specific item that has been suggested as problematic is “How long have you been thinking about the possibility of cancer recurrence?” where long-term cancer survivors may be more likely to score 4 (several years) compared to short-term survivors [17]. Nevertheless, the item may provide valuable information about the duration of FCR.

Previous studies determining cut-off scores for the FCRI-SF have primarily relied on data from samples of breast and prostate cancer patients and fewer lung and colorectal cancer patients [11, 12]. Although cancer type has been found to be unassociated with levels of FCR, younger age and shorter time since diagnosis has been found to be associated with higher levels of FCR [18]. Colorectal cancer patients are often older, and evidence is sparse within long term survivors. It is therefore important to evaluate the applicability of the clinical cut-off score in other cancer populations and at various stages of survivorship, to determine the generalizability of the FCRI-SF as a screening tool for clinical FCR.

Therefore, the aims of this study are to (1) investigate the FCRI-SF scale and item performance using factor analysis, (2) evaluate the clinical cut-off score in long-term colorectal cancer survivors with no recurrence.

## Methods

### Design

This study was guided by the COSMIN (COnsensus-based Standards for the selection of health Measurement INstruments) checklist for patient-reported outcomes [19, 20]. The data came from a national study of late effects in long-term colorectal cancer survivors in Denmark, and a randomized controlled trial (RCT) of a therapist-guided online intervention for FCR [21, 22]. Thus, the present analyses are secondary to the main purposes of the studies.

### Recruitment and data collection

#### Recruitment

All colorectal cancer survivors in Denmark were invited to participate if they fulfilled the following inclusion criteria: (a) 18 years or older; (b) diagnosed with colorectal cancer between 2014 and 2018; and (c) treated with curative intent (surgery, and/or chemotherapy, and/or radiotherapy). Participants who answered the invitation, had not had cancer recurrence, gave consent, and completed the FCRI-SF questionnaire, comprised the sample for this analysis.

#### Questionnaire data collection

Potentially eligible colorectal cancer survivors were identified through the Danish Colorectal Cancer Database (DCCG) [23]. The questionnaire was distributed digitally from REDCap [24, 25] to a personal secure digital mailbox (e-Boks). Data was gathered between May 2023 and May 2024. In this study, both questionnaire data and data from clinical assessments (see below) were used.

#### Clinical assessment of FCR

**Fig. 1:**
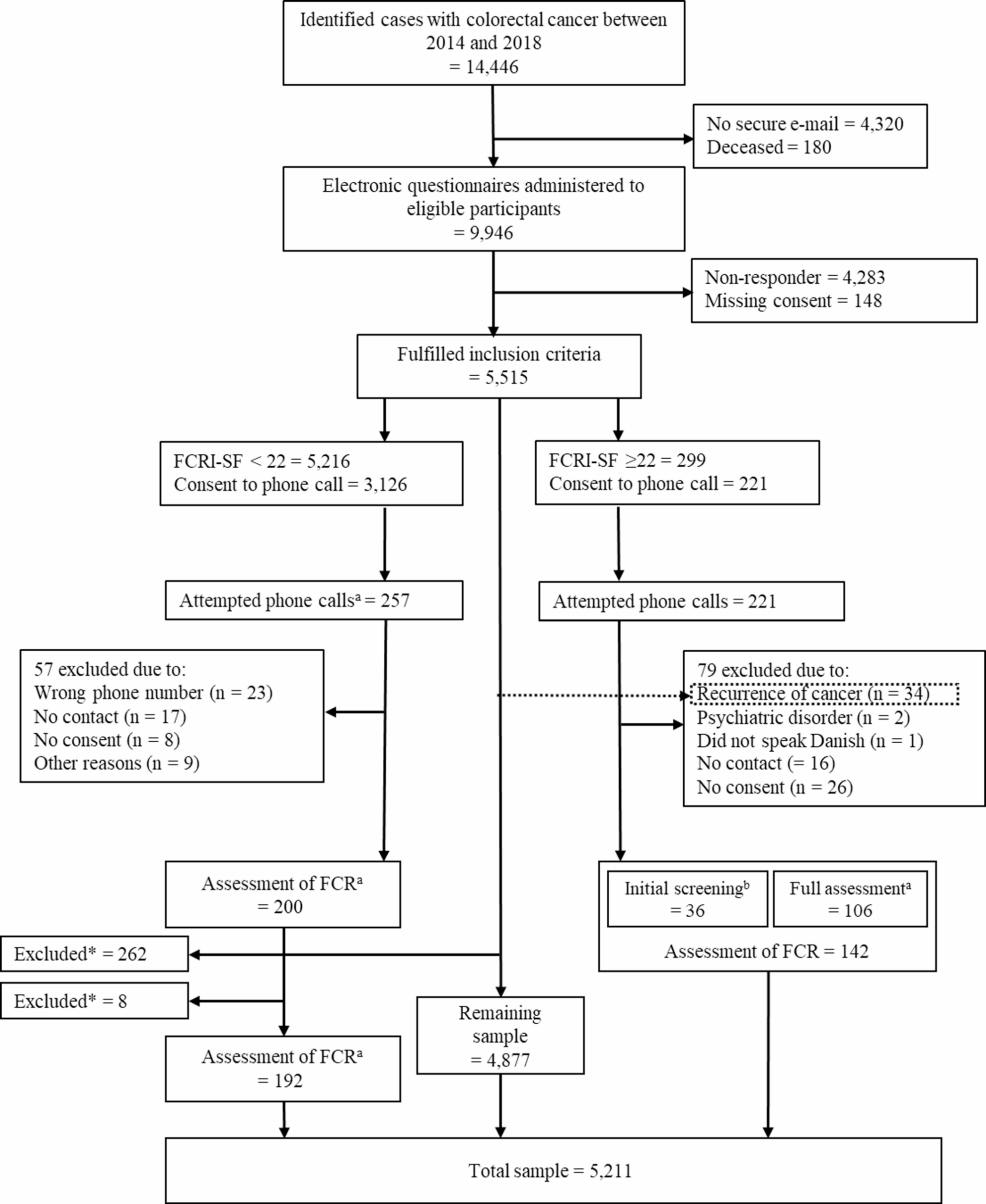
Flow chart of inclusion. FCRI-SF = Fear of Cancer Recurrence Inventory – short form; FCR = fear of cancer recurrence. Dotted line illustrates that participants with recurrence was excluded after the phone call from the total sample and from the subsample. * Based on reversed item in the FCRI-SF designed to capture inattentive automatic responses. a: conducted by psychologist. b: conducted by the project manager and did not receive full assessment due to no interest in the RCT

As part of the RCT-design participants with an FCRI-SF score ≥ 22 received a screening phone call from a psycho-oncologist, who excluded individuals reporting that FCR had no impact on their everyday life or who were already in treatment for FCR. These screenings are included in the total number of FCR assessments used for calculating cut-off (Fig. 1). All remaining participants — those expressing interest in the RCT, as well as participants with an FCRI-SF score < 22 — were invited to receive a comprehensive psychological assessment conducted over the phone by one of three psychologists within two weeks after survey completion. This assessment covered worries about recurrence, physical late effects, health anxiety, depression, panic disorder, social anxiety, generalized anxiety, obsessive-compulsive disorder, and a screening for psychotic experiences based on the Research Interview for Functional somatic Disorders (RIFD) [26] - a modified version of Schedules for Clinical Assessment in Neuropsychiatry (SCAN) [27].

FCR severity was assessed using specific items from the health anxiety section of the RIFD [26] (Supplementary material, Table S1) as several items share similarities with the consensus-based characteristics of clinical FCR [8]. These were illness-related worries, persistence of worries, and preoccupation with and hypervigilance to bodily symptoms. Other items in this section are distinctive of health anxiety like worries about contagious diseases and fear of medication. In addition, health anxiety more often involves worries about a range of possible illnesses, whereas FCR refers specifically to fears about cancer returning or progressing and may therefore represent a more realistic appraisal of threat among individuals with a prior cancer diagnosis. The mentioned overlaps were used by the psychologists to rate how much FCR disrupted usual daily activities rated as: *No impact*, *Little impact or discomfort*, *Moderate impact or discomfort*, or *Severe and invalidating impact and discomfort*. The impairment rating included emotional impact, and the psychologist assessed the overall functional impact (work, family, leisure) during the last six months. Moderate and severe ratings were considered cases of clinical FCR. The psychologists were aware of the participants’ FCRI-SF score prior to the assessment, because the RIFD uses patient screening as the starting point for assessment.

### Measures

The FCRI-SF was used to screen for FCR. It is a 9-item questionnaire rated on a 5-point scale (range 0–36, with higher scores indicating more FCR). Items 1–4 and 6 are rated 0 “Not at all” to 4 “A great deal”. Item 5 is reversed (to detect automatic responses) and answered 4 “Not at all” to 0 “A great deal”. Item 7 is rated 0 “Never” to 4 “Several times a day”, item 8 is rated 0 “I don’t think about it” to 4 “Several hours”, and item 9 is rated 0 “I don’t think about it” to 4 “Several years” (See Table 2 for item wording). The FCRI-SF has shown good test-retest reliability and convergent validity with other measures of FCR [11] and is validated in Danish [28].

#### Demographics

Demographic and clinical information regarding age, sex, cancer type, and time since diagnosis were extracted from DCCG. Participants self-reported additional demographic (education, marital status, attachment to labor market, children) and clinical information (chemotherapy and/or radiotherapy).

### Statistical analyses

Descriptive statistics included means and standard deviations for normally distributed variables, and median (range) for skewed variables. Nominal variables were summarized using frequencies and percentages.

Before analyses participants with seemingly automatic responses, defined by answering “Not at all/Never/I don’t think about it” to all items overlooking the reversed item 5, were excluded [11]. The sample was randomly split into two equal subsamples with one used for Explorative Factor Analysis (EFA) and the other for Confirmative Factor Analysis (CFA).

The FCRI-SF factor structure was examined using EFA with principal factor estimation and varimax rotation. Factor solutions with 1–3 factors were explored, and the final number of factors was determined based on the scree plot and interpretability of factor loadings.

CFA investigated fit of the theoretical one factor model using maximum likelihood (ML) estimation. Although the FCRI-SF items are ordinal, ML was chosen for pragmatic reasons related to the interpretability and comparability of model fit indices.

Several fit indices were applied: Chi-square and standardized root mean square residual (SRMR) goodness-of-fit statistics to investigate the discrepancy between observed and fitted covariance matrix, where an insignificant Chi-square test and SRMR values below 0.05 indicate good fit [29]; Comparative fit index (CFI) to assess fit relative to a null model with values from 0 to 1, where values above 0.95 indicate good fit [29]; Tucker-Lewis index (TLI) which resembles CFI but further adjusts for number of model parameters [30]; Root mean squared error of approximation (RMSEA) as an expression of lack of fit per degree of freedom of the model with values below 0.6 considered indicative of good fit [29].

To correct for the skewness introduced by the stratified sampling procedure for participation in the clinical interview, the interview sample was weighted using inverse probability to reflect the total sample. The weighting procedure was based on the mechanism of participation in the clinical interview (whether or not FCRI-SF score was over 22), as well as sex and age of participants.

Sensitivity (SE), specificity (SP), positive predictive values (PPV), negative predictive values (NPV), and misclassification rates were calculated for all possible cut-offs using weighted logistic regression with interview-based assessment criteria for FCR serving as the gold standard [31]. To establish the theoretical FCR cut-off for the FCRI-SF the maximum of the sum of SE and SP was used. A parametric probit model was estimated using maximum likelihood to create Receiver Operating Characteristic (ROC) curves and to calculate the area under the curve (AUC). FCR cases were classified as moderate and severe FCR combined based on interview data. Analyses were performed using Stata 18.5 [32].

## Results

### Participants

In total, 9,946 invitations were sent out to survivors who fulfilled the inclusion criteria. Of these, 5,515 answered the full questionnaire and provided consent. 270 participants were excluded due to automatic responses detected using the reversed item 5, leaving 5211 participants for analyses (Fig. 1). The 270 automatic responders were older with a mean age of 79 years compared to 72 years in our sample, differed in terms of sex where 75% were male compared to 57.1, in terms of education where only 14% had a long education compared to 34.3, and in terms of attachment to the labor market where 91% were retired compared to 74.7.

Assessments of FCR were conducted with 192 participants with an FCRI-SF score < 22 (0–21) and 142 participants with an FCRI-SF score ≥ 22 (22–33) (candidates for the RCT in the original study) (Fig. 1). Participant characteristics are summarized in Table 1 for the total sample and the two subsamples (FCRI-SF score < 22 and FCRI-SF score ≥ 22).

**Table 1:** Participant characteristics

Characteristic	Total sample (*N* = 5,211)	Assessments of FCR (*N* = 334)	
		≥ 22 FCRI-SF (*N* = 142)	< 22 FCRI-SF (*N* = 192)
Age mean (SD)	72 (0.1)	64 (0.9)	72 (0.6)
Gender % female (N)	42.9 (2,237)	59.2 (84)	42.7 (82)
Education % (N)			
Short education (mandatory school < = 9 y)	17 (884)	16.9 (24)	18.8 (36)
Medium education - secondary education	42.9 (2,233)	42.3 (60)	39 (75)
Long education - Higher education (> 12 y)	34.3 (1,787)	33.8 (48)	39 (75)
Other education	5.2 (270)	4.9 (7)	2.6 (5)
Missing	0.7 (37)	2.1 (3)	0.5 (1)
Employment % (N)			
Employed	22.8 (1,186)	44.4 (63)	19.8 (38)
Pension	74.7 (3,893)	52.8 (75)	76.6 (147)
Never been employed	1.1 (58)	1.4 (2)	1 (2)
Missing	1.4 (74)	1.4 (2)	2.6 (5)
Married/in relationship % (N)	72.4 (3,761)	66.2 (94)	71.7 (137)
Children % yes (N)	89.6 (4,667)	94.4 (134)	89.5 (171)
Years since diagnosis mean (range)	7.2 (4, 10)	6.9 (4, 10)	7.3 (4, 10)
Cancer type % colon (N)	66.3 (3,455)	70.4 (100)	63 (121)
Treatment % (N)			
Surgery	59.5 (3,103)	44.4 (63)	55.2 (106)
Surgery and chemo	33.8 (1,761)	49.3 (70)	37.5 (72)
Surgery, chemo, and radiotherapy	6.7 (347)	6.3 (9)	7.3 (14)
FCRI-SF mean (SD)	10 (0.1)	24.9 (0.2)	8.6 (0.4)

FCR: Fear of cancer recurrence; SD: standard differentiation; N: number; FCRI-SF: Fear of cancer recurrence inventory – short form

### FCRI-SF

**Fig. 2:**
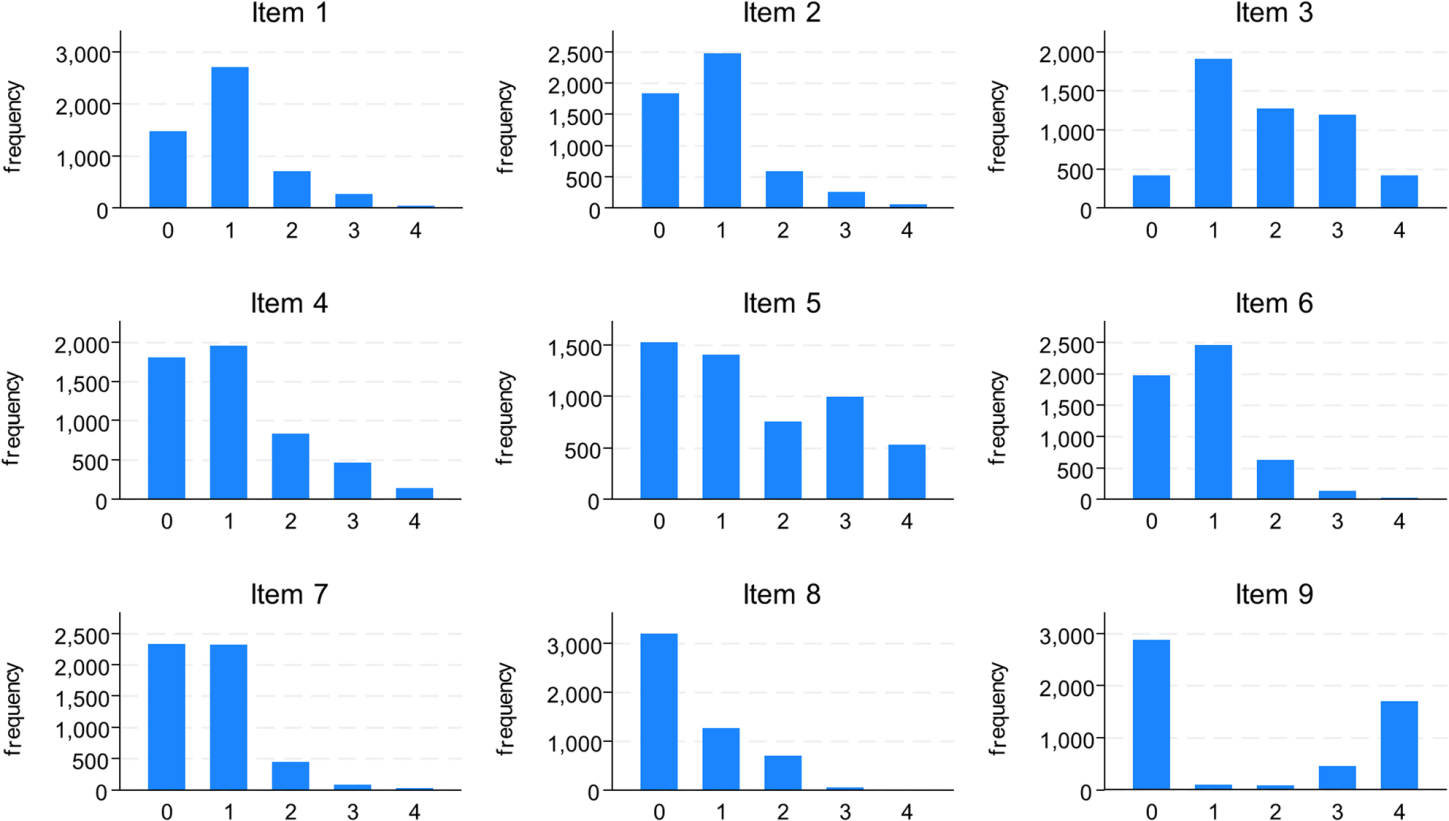
Distribution of responses for each item. For item 1–4 and item 6: 0 = Not at all, 1 = A little, 2 = Somewhat, 3 = Quite a bit, 4 = A great deal. For item 5: 0 = A great deal, 1 = Quite a bit, 2 = Somewhat, 3 = A little, 4 = Not at all. For item 7: 0 = Never, 1 = A few time a months, 2 = A few times a week, 3 = A few times a day, 4 = Several times a day. For item 8: 0 = I don’t think about it, 1 = A few seconds, 2 = A few minutes, 3 = A few hours, 4 = Several hours. For item 9: 0 = I don’t think about it, 1 = A few weeks, 2 = A few months, 3 = A few years, 4 = Several years

Therewass no missing data on the FCRI-SF, as responding to these items was mandatory. In the current study, the internal validity measured using Cronbach’s alpha αα = 0.83) was good assuming a one-factor model is valid. Distribution of responses are found in Fig. 2.

### Factor analyses

The EFA (*N* = 2606) supported a 1-factor model as assessed from the scree plot (Supplementary material, Figure S1) as well as second Eigenvalue being below 0.5. All items except one had factor loadings between 0.507 and 0.872 (Table 2). Item 5 “I believe that I am cured, and the cancer will not come back” had an EFA factor loading of 0.214.

The CFA (*N* = 2605) indicated a poor fit for a one-factor model with RMSEA = 0.155 (CI: 0.149–0.161), SRMR = 0.058, CFI = 0.872, and TLI = 0.830. All item excect one had factor loadings between 0.511 and 0.894, where item 5 had a factor loading of 0.233. Fit improvements were examined exploratively using the statistical software. Modifications indices suggested correlations between specific items (item 1 and 2, and item 7, 8, and 9) but even with these changes the fit was poor.

**Table 2:** Factor loading for each items in the FCRI-SF

Item	EFA 1 factor †	CFA
1 I am worried or anxious about the possibility of cancer recurrence	0.872	0.894
2 I am afraid of a cancer recurrence	0.850	0.868
3 I think it’s normal to be anxious or worried about the possibility of cancer recurrence	0.507	0.511
4 When I think about possibility of cancer recurrence, other unpleasant thoughts or images come to mind (death, suffering, consequences for my family)	0.681	0.682
5 I believe that I am cured, and the cancer will not come back	**0.214**	**0.233**
6 In your opinion, what is your risk of having a cancer recurrence	0.707	0.716
7 How often do you think about the possibility of cancer recurrence?	0.805	0.783
8 How much time per day do you spend thinking about the possibility of cancer recurrence?	0.748	0.724
9 How long have you been thinking about the possibility of cancer recurrence?	0.730	0.698

EFA: exploratory factor analysis; CFA: confirmatory factor analysis. Bold: factor loading < 0.5

†Factor scores from principal axis factoring

### Cut-off

Results of the assessment of clinical FCR distributed to scores below and above 22 are found in Table 3. The optimal theoretical cut-off for the FCRI-SF was 22 with a sensitivity of 76.1%, a specificity of 97.9%, a PPV of 58.4%, and an NPV of 99.1% (Table 4). Twenty-two was also the lowest possible cut-off to estimate a weighted SE for. AUC was 0.989 (CI: 0.982–0.997) (Weighted ROC-curve can be found in the supplementary material, Figure S2).

**Table 3:** Result of clinical assessment for FCR

	Clinical rating of FCR severity impact			
	No^a^	Little^a^	Moderate^b^	Severe^b^
FCRI-SF < 22	161	29	2	0
FCRI-SF ≥ 22	36	22	61	19

FCR: Fear of cancer recurrence. FCRI-SF: Fear of cancer recurrence inventory – short form

a = no clinical FCR; b = clinical FCR

**Table 4:** Results of cut-off analyses for FCRI-SF

Cut-off	Sensitivity % (CI)	Specificity % (CI)	PPV % (CI)	NPV % (CI)
22	76.1 (43.7–92.9)	97.8 (97.1–98.4)	58.4 (50-66.4)	99 (96.1–99.8)
23	67.8 (41.8–86)	98.4 (97.8–98.8)	63.1 (54-71.5)	98.7 (96.3–99.5)
24	53.6 (34.4–71.7)	98.8 (98.3–99.2)	64.7 (54.2–73.9)	98.1 (96.1–99.1)
25	45.3 (29.2–62.5)	99.3 (98.9–99.6)	72.3 (60.4–81.7)	97.8 (95.9–98.8)

NPV: Negative predictive value (probability of not having clinical FCR when score is less than cut-off score). PPV: Positive predictive value (probability of having clinical FCR when score is greater than or equal to cut-off score)

## Discussion

In this study, we examined the structure and clinical utility of the FCRI-SF in a sample of long-term colorectal cancer survivors. Consistent with previous validation studies, the FCRI-SF appeared to reflect a one-factor model based on EFA [11, 33] but this model showed poor fit when using CFA. The optimal theoretical cut-off for clinical FCR in our sample was 22. Two items, item 5 and item 9, exhibited potential psychometric problems. The results will be discussed subsequently below.

The poor model fit observed in the CFA suggests potential psychometric limitations in the one-factor model in our population of long-term survivors. A potential explanation is the heterogeneous nature of the response formats: item 1–6 assesses intensity, whereas item 7–9 assesses frequency or a time dimension. Such differences in the response options may violate assumptions of normality and introduce artificial dimensions thereby affecting fit [34]. Additional factors may include the Danish translation, where some semantic nuances might have shifted, and the overrepresentation of patients with low scores, contributing to skewed item distributions. This skewness may further challenge the assumption of normally distributed indicators underlying ML estimation. Overall, the findings may reflect structural complexities not captured by a strict one-factor model, such as method effects or latent subdimensions related to intensity versus frequency. Future research could explore these possibilities using alternative modeling approaches or by explicitly testing multidimensional structures.

In both EFA and CFA Item 5 exhibited a notably lower factor loading of 0.214. This finding is consistent with the original development study in which item 5 “I believe I am cured, and that the cancer will not come back” also had the lowest factor loading of 0.35 [11]. The poor performance of this item may be attributable to the reversed scoring, a methodological choice originally included to identify automatic response patterns [11]. Psychometric literature suggests such items can reduce model fit rather than reduce bias and that they increase the likelihood of participants making mistakes [35]. The performance may also be attributable to the item’s double phrasing - combining beliefs about being cured and beliefs about recurrence - meaning that respondents may put more weight on believing that they are cured versus believing that the cancer will not come back. This critique has previously been raised [17], with the argument that survivors cured of cancers with a high likelihood of recurrence may find it challenging to respond to this item. However, in the case of colorectal cancer, the objective risk of recurrence approximates the risk of developing a new cancer in the general population after 5–10 years [36]. Thus, in long-term survivors, a belief that the cancer will return, which does not fit with the objective risk, may be a valid indicator of FCR. Thus, while item 5 may not align optimally with the latent structure of the scale, its content may nonetheless capture a clinically meaningful manifestation of FCR in long-term survivorship with a low likelihood of recurrence, as in our study sample. Accordingly, rewording this item could potentially improve its measurement properties in future evaluations of the FCRI-SF.

Another item that attracted attention due to its U-shaped response pattern was Item 9: “How long have you been thinking about the possibility of cancer recurrence?”. The pattern indicated that participants tended to select either 0 (“I don’t think about it”) or 4 (“Several years”), suggesting that the middle response categories did not contribute meaningful information in this sample of long-term survivors. This finding supports previously raised criticisms of the item, particularly regarding its use in long-term survivorship populations [17]. Nevertheless, the factor loading for Item 9 remained acceptable, and the item continues to provide relevant information about the duration of worry in clinical contexts.

The optimal theoretical cut-off for clinical FCR in our sample was 22 aligning with the previously reported cut-off [12]. The results for SE, SP, NVP, and PPV showed that this cut-off gives a high level of certainty that individuals scoring below this threshold do not have clinical FCR. Although, it should be noted that predictive values depend on the underlying prevalence of the condition. As the prevalence of clinical FCR in this long-term survivor sample was relatively low, the PPV and NPV reported here may not generalize directly to populations with higher levels of FCR risk. Furthermore, the results for SE, SP, NPV and PPV also indicate that a proportion of individuals who screen positive for FCR on the FCRI-SF may not actually experience clinically relevant levels of fear at the assessment. This pattern is consistent with the intended function of the FCRI-SF as a screening instrument prioritizing sensitivity over specificity. Importantly, this finding underscores the clinical value of subsequent clinical evaluation, as recommended in a recent FCR guideline [37] and clinical pathway [38]. A clinical assessment allows differentiation between transient or proportionate fears of recurrence, and persistent, excessive, and functionally impairing FCR, as well as consideration of contextual and differential diagnostic factors. This distinction has direct implications for treatment planning, as not all individuals with elevated FCRI-SF scores require FCR-specific interventions. Thus, subsequent clinical assessment ensures that specialized interventions are reserved for those with clinically significant FCR.

One other study has identified a cut-off of 22 [12]. Like in our study, they used assessment data from an RCT without applying the consensus-based criteria for clinical FCR, suggesting that 22 may be an appropriate threshold for identifying survivors who might benefit from FCR-specific interventions.

### Strengths and limitations

This study has several strengths. First, the study used a population-based, nation-wide sample of colorectal cancer survivors from a universal and tax-funded health care system, which strengthens generalizability in this specific cancer population and reduces selection bias. Accordingly, as colorectal cancer is most often diagnosed above age 50 [39, 40], the mean age in our sample was high. This may limit the generalizability to other cancer populations but contributes important knowledge about older cancer survivors – an under-researched group despite the increasing prevalence of cancer survivorship in this age group [41, 42]. Second, the assessments were thorough and conducted by psychologists with extensive experience in digital assessment of health anxiety strengthening the validity of the clinical assessment of FCR. Third, the use of weighted logistic regression and ROC analysis increased the precision of the cut-off and accounted for the stratified sample.

One limitation concerns the choice of estimation method in CFA. We faced a trade-off between potential violations of the normality assumption for the ML method and challenges in interpreting the fit indices from the WLSMV method. We deemed the latter to be the larger threat. Other limitations pertain to the secondary nature of the study. First, the sample was already split into two groups using 22 as the cut-off, and the psychologists were aware of the participants’ FCRI-SF scores prior to conducting the assessments. This was done to identify cases eligible for participation in the RCT. However, this approach may have introduced verification bias when validating the cut-off, by increasing the likelihood that the clinical evaluation corresponded with the questionnaire scores, thereby potentially inflating estimates of diagnostic accuracy, including the AUC. To partly mitigate this risk, we included a randomly selected subgroup of 192 participants scoring below 22, in addition to 142 participants scoring above 22. By assessing participants from both sides of the threshold, the analysis captures a range of FCR severity, which strengthens the validity of the cut-off despite the initial group-based sampling. Nevertheless, the number of participants scoring below the cut-off may still have been insufficient to capture the full variability of low FCR levels, and some degree of verification bias cannot be ruled out.

Second, due to the RCT design, the assessments followed two different procedures, representing a significant limitation to the study. Participants with a score of 22 or above were first evaluated by a psycho-oncologist regarding the impact of FCR on their daily life before undergoing the full assessment. In addition, twenty-six participants did not provide informed consent to the primary investigator to participate in the RCT, and it is therefore unknown whether they experienced FCR. Third, the clinical assessment conducted in this study was not originally designed for a validation study. We assessed illness-related worries, rumination/persistence, preoccupation with bodily symptoms, tendencies to seek medical advice, searching online for symptom-related information, and function level and impact of FCR. We did not adhere to specific criteria for clinical FCR, as the inclusion in the RCT did not rely on this. Our assessment was based on an assessment of health anxiety, which may have influenced the validity of the gold-standard. However, the questions reflect the consensus-based criteria for FCR: high levels of preoccupation with symptoms, high levels of worry about recurrence that are persistent, and hypervigilance to bodily symptoms [8]. Nonetheless, FCR has been emphasized as a distinct clinical construct separate from health anxiety requiring specific assessment [4, 8, 43, 44]. Though this discussion is ongoing, some authors argue that FCR and health anxiety reflect the same underlying pathology [45, 46], it is a limitation to our study that the assessment relied strongly on questions aimed addressing health anxiety. Previous studies investigating cut-offs on the FCRI-SF used either a biopsychosocial assessment addressing cognitive and behavioral features of FCR as part of an RCT study [12] or the Structured Interview for Fear of Cancer Recurrence (SIFCR) [12, 47]. A recent paper introduced a new interview addressing the agreed upon criteria of FCR [8, 48]. Future assessments used as a gold standard for FCR should consider adopting one of these interviews.

## Conclusion

This study aimed to investigate the factor structure of the FCRI-SF and determine the clinical cut-off in long-term colorectal cancer survivors without recurrence. The factor analyses supported a one-factor model, but with poor CFA model fit. The poor fit may be explained by the heterogeneity of the response options and two problematic items. Item 5 demonstrated suboptimal psychometric properties and may benefit from rewording. In long-term survivors, item 9 may be less informative, as responses tend to cluster at two extremes: either no FCR or persistent worries lasting several years. Consistent with previous research, the optimal theoretical cut-off point for identifying clinical FCR was 22, primarily reflecting a high level of certainty that individuals scoring below this threshold are unlikely to have clinically FCR.

## Supplementary Information

Below is the link to the electronic supplementary material.

Supplementary Material 1

## Data Availability

The dataset generated during this study is available from the corresponding author upon request.

## Abbreviations

FCR: Fear of cancer recurrence
FCRI-SF: Fear of cancer recurrence inventory – short form
HA: Health anxiety
COSMIN: COnsensus-based Standards for the selection of health Measurement INstruments
DCCG: Danish colorectal cancer database
RCT: Randomized controlled trial
RIFD: Research interview for functional somatic disorders
SCAN: Schedules for clinical assessment in neuropsychiatry
EFA: Exploratory factor analysis
CFA: Confirmatory factor analysis
SRMR: Root mean square residual
CFI: Comparative fit index
RMSEA: Root mean squared error of approximation
SE: Sensitivity
SP: Specificity
TLI: Tucker-Lewis index
ROC: Receiver operating characteristic
PPV: Positive predictive value
NPV: Negative predictive value
AUC: Area under the curve
SIFCR: Structured Interview for fear of cancer recurrence
