# Supplementary material for: Validation of the fear of cancer recurrence inventory-short form in long-term colorectal cancer survivors
Source: J Patient Rep Outcomes. 2026 Apr 18;10:110. doi: 10.1186/s41687-026-01051-y (PMC13341998; doi:10.1186/s41687-026-01051-y)
Supplement: Supplementary file 1 — Supplementary Material 1 [file 41687_2026_1051_MOESM1_ESM.docx]

**Supplementary material**

| Table S1. Assessment health worries | | |
| --- | --- | --- |
| Health worry/  behavior assessed | Question | Rating |
| Illness worries | *Are you often worried that there might be something wrong with your health, that you might have a serious illness?* | 0: No particular concern  1: Mild to moderate concern, but no significant interference with daily life  2: Severe concern involving medical consultations, examinations, persistent self-medication, or pronounced difficulty in daily life |
| Rumination | *If you have thoughts about possibly being ill, do you find it difficult to get the thought out of your head?* | 0: No particular preoccupation.  1: Light to moderate preoccupation, but no significant interference with daily life.  2: Severe preoccupation with significant interference with daily life. |
| Preoccupation with bodily symptoms | *Are you generally occupied with how your body feels and what is happening in it?*) | 0: No particular preoccupation.  1: Light to moderate preoccupation, but no significant interference with daily life.  2: Severe preoccupation with significant interference with daily life. |
| Preoccupation with health information | *Are you generally preoccupied with health-related topics in newspapers, magazines, TV, or on the internet?* | 0: No particular preoccupation.  1: Light to moderate preoccupation, but no significant interference with daily life.  2: Severe preoccupation with significant interference with daily life. |
| Suggestibility | *If you hear about a disease, do you become afraid that you might have that disease yourself? Do you avoid reading about illnesses to prevent getting scared?* | 0: No particular impact.  1: Light to moderate impact but no significant interference with daily life.  2: Severe impact with worries and interference with daily life |
| Fear of contamination | *Are you often afraid of getting infected or contracting a disease from others by being in contact with them or touching dirty objects (e.g., a toilet seat)?* | 0: No particular fear.  1: Light to moderate fear but no significant interference with daily life. 2: Severe fear with interference with daily life. |
| Ability to accept reassurance from a doctor | *Has the doctor's reassurance convinced you that there is nothing wrong?* | 0: Accepts the doctor's reassurance.  1: Has been unable to maintain the doctor's reassurance on one or more occasions.  2: Has been unable to maintain the doctor's reassurance most of the time. 3: Has consistently been unable to accept the doctor's reassurance or explanations. |
| Fear of taking medication | *Are you generally comfortable taking medication, or do you have concerns about any potential side effects it might have?* | 0: Normal level of comfort/discomfort.  1: A certain level of medication fear or some degree of inappropriate medication administration.  2: High level of fear and/or lack of compliance. |
| FCR severity | *How much have the mentioned concerns about fear of recurrence, overall disrupted your usual daily activities?* | 0: No impact.  1: Little impact or discomfort.  2: Moderate impact or discomfort.  3: Severe and invalidating impact and discomfort. |

Figure S1. Scree plot of eigenvalue after factor.

Figure S2. Weighted ROC-curve
